# Relationship Between Virtual Pharmacology Lecture, Attendance and Performance: Personal Experience With Veterinary Students of the University of BUEA, Southwest Cameroon

**DOI:** 10.1002/prp2.70118

**Published:** 2025-08-03

**Authors:** Saganuwan Alhaji Saganuwan, Manchang Tanyi Kingsley

**Affiliations:** ^1^ Department of Veterinary Pharmacology and Toxicology College of Veterinary Medicine, Federal University of Agriculture Makurdi Benue State Nigeria; ^2^ Department of Veterinary Medicine, Faculty of Agriculture and Veterinary Medicine University of BUEA Buea Southwest Cameroon

**Keywords:** advantage, Cameroon, disadvantage, online lecture, pharmacology, University of Buea, veterinary medicine, zoom

## Abstract

Risk and cost of transportation and other unforeseen circumstances can hamper in‐person pharmacology lectures with students. Hence, the study is aimed at identifying the advantages and disadvantages of pharmacology lectures via Zoom. A crossover design was adopted for the study that involved a total of 151 students of veterinary medicine, which comprised students of 400 level (52), 500 level (52) and 600 level (47) who participated six times in the study for one semester. All the lectures were delivered online. The levels and average numbers of online lecture participants were 400 level (22.3), 500 level (22.8) and 600 level (21.3) for Principles of Pharmacology, Applied Pharmacology, and Therapeutics, respectively. The findings showed no significant difference in online connection (*p* > 0.05) between 400, 500, and 600 level students. The numbers of students that passed Principles of Pharmacology (15), Applied Pharmacology (13) and Therapeutics (14) showed that the level of study and performance on the tests were connected with lecture attendance. Fourty‐four percent attended the lectures, whereas 56% were absent. However, 27.8% of all the students passed the tests, whereas 72.2% failed the tests, respectively. The highest number of feedbacks (*p* < 0.05) was received from 500 level students (32), as compared to 400 level students (24) and 600 level students (17), respectively. The study has shown that online pharmacology lectures can serve as an alternative to in‐person lectures, though associated with technical problems. However, many students showed much enthusiasm and praised the lecturer for his frantic effort in delivering the lectures, cordial lecturer/student relationship, and his objectivity toward class. The failure of power supply, problems of coordinating courses, zoom meeting network issues, insufficient laptops and phones, as well as the problem of connection with the course lecturer for the online lectures are disadvantages. Online lecturer/student relationship, objectivity of the lecturer, and lecturing methods can boost students' morale and interest in online lectures.

## 1 Introduction

Pharmacology is a fundamental medical discipline that is difficult and counterintuitive to learn. Therefore, navigation toward understanding the course may be troublesome, and once the threshold to understanding is crossed, learners can experience a shift in their ways of thinking and practicing. The concepts such as pharmacokinetics, pharmacotherapeutics, drug mechanism of action, drug receptors interactions, drug terminology and nomenclature, and signaling pathways align with key attributes of the concept, which are transformative, integrative, and troublesome [1], suggesting the need to keep learning when necessity arises [2]. The choice of veterinary medicine as a course of study is dependent on the student interest, which can be promoted by student‐lecturer relationship. The interest in veterinary medicine is correlated with interest in pharmacology [3]. Zoom technology has been reported to enhance positive outcomes among diverse groups of medical students. It encourages higher education in faraway areas, invariably reducing workloads for teachers [4]. Zoom teaching is highly effective and enjoyable and improves achievement in learning, though it requires preparation, presentation and practice (PPP), evaluation and expansion (EE) [5]. Understanding of teaching by students via zoom learning could be up to 72.5% as observed during the COVID‐19 pandemic. The effectiveness of zoom learning was based on learning environment, student interaction, access, carrying capacity, and managing application in zoom [6]. Hence, online teaching via zoom facilitates a higher level of academic achievement and interest among students than in‐person lecture methods [7]. Therefore, zoom technology is quite viable for emergency remote lectures, though it requires collaborative interaction, flexibility, adaptability, and reliability of performance. The disadvantages of zoom learning are problems of connectivity and complexity of features, underscoring the importance of the most suitable online learning platform [8]. Zoom is easy to create with minimal administrative requirements [9] and solves problems of security challenges associated with learning [10]. Teaching of pharmacology requires integrating knowledge of physiology, biochemistry, microbiology, and pathology; therefore, virtual reality can change pharmacology education to engaging and effective learning [11]. Blended learning promotes engagement, satisfaction, and outcomes of learners [12]. Technology‐inspired engagement can strengthen learning and retention of pharmacology. Early exposure to pharmacology from preclinical to clinical can enhance pharmacology learning in medical curricula [13]. In view of the necessity for virtual learning during epidemics, cost of traveling, and traveling logistics, the study is aimed at investigating the advantages and disadvantages of teaching pharmacology to students of Veterinary Medicine, University Buea, Southwest Region of Cameroon.

### 1.1 Statement of the Problem

A number of veterinary students are either withdrawn or repeating undergraduate IV, V, or VI, while others drop out because of failing pharmacology courses. More so, the risk and cost of transportation and other unforeseen circumstances could hamper in‐person pharmacology lectures with students. Hence, there is a need to find out the cause of students' failures with the intent of proffering lasting solutions, bearing in mind that online lectures could be responsible for their failures.

### 1.2 Purpose of the Study

- To determine the level of online lecture attendance among 400, 500, and 600 level students offering Principles of Pharmacology, Applied Pharmacology, and Therapeutics, respectively.
- To determine the advantages and disadvantages of online lecture attendance among 400 500, and 600 level students of the University of Buea offering the pharmacology courses.
- To determine the performance of the 400, 500, and 600 level students at the Principles of Pharmacology, Applied Pharmacology, and Therapeutic tests after the online lectures.

### 1.3 Research Questions

- Does online lecture affect attendance of 400, 500, and 600 level students in Principles of Pharmacology, Applied Pharmacology, and Therapeutics, respectively?
- Are there advantages and disadvantages that affect online lectures in the pharmacology courses?
- Does online lecture affect 400, 500, and 600 level students' performance at the Principle of Pharmacology, Applied Pharmacology, and Therapeutics tests?

### 1.4 Research Hypotheses

- The null hypothesis states that online lecture does not affect attendance of students in Principles of Pharmacology, Applied Pharmacology, and Therapeutics.
- The null hypothesis states that there are no advantages or disadvantages in online pharmacology lectures.
- The null hypothesis states that online lectures do not affect students' performance at the pharmacology courses.

### 1.5 Significance of the Study

The study is expected to reveal the level of students' attendance, merits and demerits of online lecture and students' performance at the pharmacology courses after online lectures.

### 1.6 Scope and Limitation of the Study

The study covered 400–600 level undergraduate students that comprised male and female students who offered pharmacology courses.

## 2 Materials and Methods

### 2.1 Research Design

The study was designed to determine the advantages and disadvantages of teaching pharmacology to veterinary students of the University of Buea, Southwest Region of Cameroon. Specific Measurable Achievable, Relevant, and Time‐bound (SMART) framework was adopted for achieving clear focused learning objectives. Structured course design, robust student support, and engaging pedagogy are encompassed to ensure effective learning outcomes.

### 2.2 Area of the Study

The area of the study was the University of Buea, Southwest Cameroon located in Fako division at altitude (900 m) on the slope of Mount Cameroon, latitude 4°14″ north of the equator and longitude 9°20″ east of the Greenwich [14].

### 2.3 Population of the Study

A total of 151 students were sampled from the Department of Veterinary Medicine, Faculty of Agriculture and Veterinary Medicine, University of Buea, Cameroon. The students comprised males and females from different religions and ethnic backgrounds.

### 2.4 Sample and Sampling Techniques

Cross over design was adopted for the study [15]. Veterinary students of the University of Buea, Cameroon, were selected for the study; their populations and the levels were 400 (52), 500 (52) and 600 (47), respectively. The courses taken via zoom meeting were Principles of Pharmacology (400 level), Applied Pharmacology (500 level) and Therapeutics (600 level), respectively. The lectures scheduled for 11 a.m. to 9 p.m. started on October 9, 2024 and ended on November 2, 2024. All the lectures were made compulsory for students and presented via zoom by Prof. Saganuwan Alhaji Saganuwan. Dr. Manchang Tanyi Kingsley presented the list of the students that offered the courses, outlines of the three courses, times, and days of the lectures. All the coordinators for the three courses were given the opportunity to schedule times and days for the lectures. The links for the zoom meetings were given 1 to 2 days before or a few minutes before the meetings or after the lecturer had connected online. Each lecture was scheduled for 2 h, though usually broken by the zoom coordinator after 40 min, and reconnection took place 10 min thereafter. The lectures lasted on many occasions for 1 h 30 min. The students usually connected online using their laptops and phones. They were usually encouraged to ask questions and give their feedback at the end of all the lectures for evaluation and judgment. In‐person tests were administered at the end of the lectures to students of all the levels.

### 2.5 Method of Data Collection and Statistical Analyses

The numbers of students that registered and attended the zoom lectures, times and dates of lectures as well as the comments made after the lectures were collated and analyzed using non‐parametric statistics. The data generated were presented in tabular form. Chi square was used to analyze the data at the 5% level of significance [16].

## 3 Results

Lecture schedules, numbers of the students that registered and attended zoom lectures, and comments of the students about lectures on the course, Principles of Pharmacology, are presented in Table 1. Fifty‐two registered students were expected to attend six lectures scheduled for 2 h each. Out of 52 registered students, only an average of 22.3 attended the lectures. Positive comments made by the students on the lectures were: very interesting; alright; really great; we are interested in asking questions; the class was superb; the class was nice; the comments were positive; and explain the different dose phenomena. However, the negative comments were: the lecture was fast; note needed; generate more time for zoom lecture; video lecture needed; we may not be able; Microsoft meets and script may be used; terms are new, calculations not understood; I am waiting for the host to start the meeting; Saturday lecture coincided with another zoom lecture; no light in Buea; peoples' phones are off; and can we have the class on Monday?

**TABLE 1:** Lecture schedules, registered students, zoom lecture attendance and comments of the students after lectures on principles of pharmacology.

Level	Date	Day	Time	Number of students registered	Number of students connected	Positive comments	Negative comments	Responses of lecturer to the comments
400	9/10/24 15/10/24 17/10/24 19/10/24 22/10/24 21/11/24	Wednesday Tuesday Thursday Saturday Tuesday Saturday	11 am‐1 pm 3 pm‐5 pm 10 am‐12 pm 3 pm–5 pm 2 pm–4 pm 12 pm–2 pm	52 52 52 52 52 52	24 17 31 18 24 20	Interesting, alright, really great, interested in asking questions after lecture, I will inform them, superb, the class was nice, the comments are positive, explain the different dose phenomena, was very interesting, connected to my laptop, the lecture was great	Note needed, generate more time, video lecture needed, we may not be able, Google meet or skype maybe used, new terms, calculation not understood, wait for the host to start the meeting, Saturday lecture coincided with another zoom lecture, no light in Buea, people's phone are off, can we have the class on Monday?, network was the issue	Question can be asked, asking question makes me understand their level of comprehension, why was your phone off?
Total		6 days	12 h	312	134	_	_	_
Average				52.0 ± 0.0	22.3 ± 2.1	_	_	_

Table 2 shows the lecture schedules, registered students, zoom lecture attendance, and comments from the students on Applied Pharmacology. Fifty‐two students registered for the course and were expected to attend the six zoom lectures, but only an average of 22.8 attended the zoom lectures. The positive comments made by the zoom students were: very interesting; thank you Prof for the lecture; it was interesting; I got lost and was able to get back on track; the class was good; he was explaining well; today's lecture was very interesting and interactive; I really enjoyed it; the lecture was good; more examples needed; more interesting; thank you for the spellings; it helps a lot; understood better now; the lecture was nice today; I followed up well; and the class was really interesting. However, the negative comments were: network issues; too fast; many things to assimilate; note needed for easy interaction; a bit fast; I couldn't jot down much; too much to grasp at once; network problem didn't allow us to connect; we tried several times but to no avail; is it possible to use slides; the terms and your lecture are really new; send notes so that we can master the diseases and drugs; I did not understand pacemaker and drug types; and students are pairing up to use phones, being the reason why online attendance is low.

**TABLE 2:** Lecture schedules, registered students, zoom lecture attendance, and comments of the students after lectures on applied pharmacology.

Level	Date	Day	Time	Number of students registered	Number of students connected	Positive comments	Negative comments	Responses of lecterer to the comments
500	16/10/24 18/10/24 19/10/24 22/10/24 24/10/24 25/10/24	Wednesday Friday Saturday Tuesday Thursday Friday	1 pm‐3 pm 3 pm‐5 pm 1 pm‐3 pm 3 pm‐5 pm 3 pm‐5 pm 3 pm‐5 pm	52 52 52 52 52 52	26 28 22 18 20 23	Very interesting, thank you Prof for the lecture, it was interesting, I got lost and was able to get back on track, the class was good, he was explaining well, today lecture was very interesting and interactive, I really enjoyed it, the lectures are good, more interesting, more examples are needed, thank you for the spellings, it helps a lot, understood better now, the lecture was nice today, I followed up well, the class was really interesting	Network issues, too fast, many things to assimilate, note needed for easy interaction, a bit fast, I couldn't jot down much, too much to grasp at once, network didn't allow us to connect, we tried several times but to no avail, is it possible to use slides?, the terms and your lecture are really new, send note so that we can master the diseases and drugs, I didn't understand pace maker, and drug types, students are pairing up to use phones, the reason why online attended is low	Note will be given, I need more time to prepare a highly comprehensive and concise note, slides may not be detailed as note, be patients you will receive comprehensive notes
Total		6 days	12 h	312	137	**_**	**_**	**_**
Average				52.0 ± 0.0	22.8 ± 1.5	**_**	**_**	**_**

Lecture schedules, numbers of registered students, zoom lecture attendance, and comments of the students after the lecture on Therapeutics are presented in Table 3. Forty‐seven students registered for the course and were expected to attend the lectures. However, out of 47 registered students, only an average of 21.3 attended the zoom lectures. The comments made by the students were: lecture was good; interesting; it was ok until the point of antiseptics; today was better I could jot at least something; may we be copying as you teach?; my network was ok; the lecture was cool; the class was good I actually enjoyed; the lecture was good; and he teaches well. Nevertheless, the negative comments were: we were using Teams before; network issues; notes needed for better understanding; I do not understand because he is fast; zoom uses so much data; network is very poor; can we try Microsoft Teams rather than zoom?

**TABLE 3:** Lecture schedules, registered students, zoom lecture attendance, and comments of the students after lectures on therapeutics.

Level	Date	Day	Time	Number of students registered	Number of students connected	Positive comments	Negative comments	Responses of lecturer to the comments
600	17/10/24 18/10/24 21/10/24 24/10/24 25/10/24 31/11/24	Thursday Friday Monday Thursday Friday Thursday	7 pm‐9 pm 7 pm‐9 pm 5 pm‐7 pm 5 pm‐7 pm 5 pm‐7 pm 5 pm‐7 pm	47 47 47 47 47 47	24 21 13 22 21 27	Lecture was good, interesting, I was ok till the point of “antiseptic,” today was better I could jot at least something, may we be copying as you teach, my network was ok, the lecture was cool, the class was good I actually enjoyed it, the lecture was good, he teaches well	We were using Teams, network issues, notes needed for better understanding because he is fast, zoom uses so much data, network is very poor, can we try Microsoft Teams rather than zoom?, we prefer Teams	Zoom was better this morning, you shouldn't worry, I will do the needful to get the best out of our efforts, we will schedule class earlier next week to avoid network disturbance, noted, I used to buy enough data, may be it will be better, we prefer Teams
Total		6 days	12 h	282	128	**_**	**_**	**_**
Average				47.0 ± 0.0	21.3 ± 1.9	**_**	**_**	**_**

Frequencies of the students that registered and attended the zoom lectures are presented in Table 4. A total of 151 students registered for the three courses. However, 43.7% (66) attended the lectures, whereas 56.3% (85) were absent at the lectures, respectively.

**TABLE 4:** Frequencies of the students that attended the zoom lectures.

Courses	Level	Number of students absent	Number of students attended	Marginal Total
Principles of pharmacology	400	30	22	52
Applied pharmacology	500	29	23	52
Therapeutics	600	26	21	47
Grand Total		85	66	151

Table 5 shows frequencies of the positive and negative comments made by the students that attended the zoom lectures. A total of 73 comments that comprised positive (38) and negative (35) comments were made (*p* < 0.05). Comments (32) made by the students who attended zoom lectures on Applied Pharmacology were significantly higher (*p* < 0.05) than comments made by the students who attended zoom lectures on Therapeutics (17). However, comments (24) made by the students that offered Principles of Pharmacology were significantly higher (*p* < 0.05) than comments made by those that offered Therapeutics. Nevertheless, positive comments (16) were significantly higher (*p* < 0.05) for Applied Pharmacology as compared to Therapeutics (10) and Principles of Pharmacology (12), whereas negative comments were significantly higher (*p* < 0.05) for Applied Pharmacology (16) as compared to Principles of Pharmacology (12) and Therapeutics (7), respectively. Numbers of the students that failed/passed, registered, and attended the Zoom lectures are presented in Table 6. Out of 52 students that sat for the Applied Pharmacology exam, only 13 passed and 39 failed, respectively. Principles of Pharmacology was passed and failed by 15 and 37 students, respectively. Meanwhile, 14 and 33 students passed and failed Therapeutics, respectively (Table 6).

**TABLE 5:** Frequencies of the positive and negative comments made by the students that attended the zoom lectures.

Courses	Positive comments	Negative comments	Marginal total
Principles of pharmacology	12	12	24
Applied pharmacology	16	16	32
Therapeutics	10	7	17
Total	38	35	73

**TABLE 6:** Performance of students at tests at the end of zoom lectures.

Courses	Pass (≥ 50%)	Fail (< 50%)	Marginal total
Principles of pharmacology	15	37	52
Applied pharmacology	13	39	52
Therapeutics	14	33	47
Marginal total	42	109	151

## 4 Discussion

The feasibility of zoom lectures at the specific range of time (11 a.m.–9 p.m.) during the study period shows that zoom teaching can serve as an alternative or complementary to in‐person teaching. However, connection by the registered students (Tables 1, 2, 3, 4) was generally low. This could be attributed to failure of power supply, network problems, limited number of laptops, phones, or desktops to use, and the costliness of the zoom data, as confirmed in the feedback given by the students. Many students attested that the lectures were interesting, alright, really great, superb, nice, good, well, interactive, enjoyable, ok, better, and cool, and thanked the lecturer during the lectures. Our findings agree with the report indicating that zoom learning teaches students how to log in and post to the chat, especially questions, reactions, and contributions [17]. Therefore, zoom's effectiveness depends on personal motivation, teacher inventiveness, availability of device support, atmosphere, and cost at the private and public levels [18], suggesting zoom teaching and learning could be enhanced by incorporating multimedia principles with the cognitive theory of multimedia learning [19], thereby increasing the effectiveness and productivity of teachers and learners [20]. Zoom meeting supports distance learning by being virtual, verbal, saving time, and being assessed repeatedly [21]. Teaching pharmacology online is necessary and should be tried and practiced because there are lessons to learn from it. Careful design and creativity in zoom teaching can bridge the gap between in‐person and visual learning, especially when necessity arises [22]. Our findings disagree with the report indicating that communication, interaction, and group dynamics are less effective at online seminars. However, the zoom lecture is favored for time saving and flexibility. The disadvantages of online seminars are reduced student interest, discussion among students, poor communication, technical problems with internet connection, and sound quality, as testified by the students. Better group dynamics, smaller groups, clearer guidelines, and better chat functionality might improve online seminars [23].

Moderate participation exhibited by all the levels of students shows that the students have enthusiasm for Principles of Pharmacology, Applied Pharmacology, and Therapeutics. Saganuwan and Egbe‐Okpenge had earlier reported the correlation of the interest between pharmacology and veterinary medicine, and the interest can be further motivated by a good relationship between students and the lecturer [3]. Khurshid et al. had earlier reported that pharmacology could be difficult and that navigation toward understanding the course may be troublesome; hence, there is a need to cross the threshold to understanding. The threshold concepts to cross are the components of the Principles of Pharmacology [1]. This underscores the need for more time to enable students to improve on their interest in pharmacology. Hence, the lecturer made an effort to reduce the speed of the lectures, explained the new terms extensively, and encouraged the students to ask questions during lectures. However, there was a need for students to have laptops or handsets to enable them to connect with the lecturer. Comprehensive, concise, and detailed lecture notes on topics taught were given to all the students of 400, 500, and 600 levels for reading. Zoom performance is comparable to Microsoft Teams, reported to be effective, and enhances classroom organization and facilitates teaching [24]. Therefore, content‐specific knowledge allows the lecturer to perceive information that maximizes memory. Hence, general and content‐specific problem‐solving approaches are necessary for Zoom lectures, which require analysis, evaluation, and self‐monitoring [25], indicating that need, time, place, and circumstance have significant impacts. So, orderliness, shared goals, and catalyzed activity are needed to empower zoom students [26], pointing to the companionship benefits [27]. The success of 15 students at the Principles of Pharmacology test in relation to zoom average lecture attendance (18), Applied Pharmacology (13 passed; 23 attended) and Therapeutics (14 passed; 21 attended) agrees with the report indicating that online lecture attendance has the likelihood of improving the success rate [28], connoting that in‐person attendance may no longer be a good marker for performance [29]. Hence, regular monitoring can identify students who may require additional academic support [30]. Differences in the level of attendance, lecture time, and number of lecture attendances have a significant positive impact on the performance of students. Therefore, students that do not attend in‐person lectures have three times the chance of failing tests, pointing to the need for monitoring and motivating students lecture attendance [31, 32]. Students' goal orientation may identify students who are less likely to attend in‐person lecture [33], despite the report that digital lecture attracts higher attendance rate as compared to traditional in‐person lecture [34]. Not liking the lecture is the most common reason for not attending lectures that determine academic outcomes [35]. So, curricular changes that encourage consistent lecture attendance could improve learning outcomes as observed in Pharmacology examination [36]. Supplemental instruction may increase academic rigor and benefit students during test or examination [37]. Nevertheless, relationship between gender, students' attendance, and academic performance for clinical and preclinical teaching activities is necessary [38]. The use of graded attendance policies for increasing class attendance may be necessary [39] for better performance at examinations or tests [40]. Our findings disagree with the report that virtual students could attend lectures more than in‐person students, yet perform worse because virtual students are receptive and feel less competitive when compared to in‐person students [41, 42]. Passing the tests by many students connotes that monitoring online lectures may identify students at risk of poor performance [43], as there is a relationship between student performance and class attendance [44]. Therefore, there is a need for inclusive Pharmacology education that comprises a positive mindset, diverse culture, history, relevance, sense of belonging, and representation. Understanding neurodiversity, disability, educational and socioeconomic background, genetics, ethnicity, race, sex, and gender improves education for all learners [45]. However, pedagogical content knowledge, teaching strategies, more time for curricula, and the use of models to understand drugs are required in pharmacology subject matter. Real challenges for pharmacology teaching are new educational insights and scientific development in teaching and learning [46], such as computer simulation and other models as alternatives to Pharmacology education [47]. The challenges posed by the students are school exclusion, sickness absenteeism, and unexcused absenteeism, which have a negative impact on students' academic achievement. Unexcused absenteeism is more harmful throughout the academic year, while sickness absenteeism is more harmful at the beginning of the academic year [48]. Lack of constant access to academic staff who are experts in their discipline can make students lose guidance, quality feedback, and support [49, 50]. The complex and abstract nature that requires education to bridge theory with practical application and diverse learning styles has made the teaching of pharmacology unique [51]. Therefore, the present study shows that online learning of pharmacology courses could improve the understanding of the course contents and the performance of students at tests. This is corroborated by the report indicating that humor, instructional strategies, motivation, student engagement, and problem‐based learning improve learning outcomes in the pharmacology course [52].

The registration‐attendance‐pass ratio for Principles of Pharmacology (52:22.3:15), Applied Pharmacology (52:22.8:13) and Therapeutics (47:21.3:14) shows that the expanding field of pharmacology can make pharmacology education difficult, suggesting that the students have perceived the course to be hardtolearn. Therefore, technology‐inspired student engagement can strengthen pharmacology learning (13), using core concepts such as big ideas, difficulty, and endurance that are useful to solve problems and applicable across content [53]. The present novel discovery is that, as the number of course registrations increases, the online lecture attendance and test performance decrease, denoting that pharmacology teaching requires tips such as avoidance of information overload, provision of handouts, beginning of lectures with a case scenario, arrangement of educational content in graphical shows and reviews of lectures, use of illustrations, interlinking of pharmacological concepts, pathophysiology, and evolutionary concepts of diseases, related historical anecdotes, the use of generic names of drugs, use of available social media, and a pharmacology lecturer has to be a lifelong learner [54]. Sustained content recall and application of pharmacology knowledge could be achieved by active learning [55] which enhances rational, safe prescription and improves patient care [56]. However, collaborative learning, computer‐assisted modules, and role plays could improve motivation, skill development, and knowledge retention [57]. Computer‐AssistedLearning (CAL) with audio‐visual aids has a great impact on student learning [58]. Positive and negative feedback received by the lecturer from the students are corroborated by the report indicating that systemic and regular feedback from the learners could improve the quality of education as clinically oriented teaching has a positive impact on the study of pharmacology [59], connoting that a constructive learning could maximize the impact of pedagogical intervention in the teaching of pharmacology [60]. Hence, a dynamic and interactive learning environment creates student autonomy, enriches students autonomy, educational experience, and learning [61]. Creativity in the classroom brings a powerful emotional value to the subject that is connected with positive and constructive memories [62], suggesting that the flipped classroom approach could be adopted for teaching pharmacology to ensure students good performance [63].

## 5 Conclusion

Zoom pharmacology lecture was associated with low network connection by students. Lecturer‐student relationship, mode of teaching pharmacology, course taught, technical issues, coordinators of the courses and zoom meeting, lack of power supply, lack of adequate laptop/phones to connect, and method of teaching can affect pharmacology zoom lecture. Hence it is advised that the zoom lecturer should be highly objective in asking the students to give their feedback at the end of every lecture, to enable him to know the problems encountered during the zoom lecture. Subjectivity of the lecturer may frustrate the students and make the zoom lecture less interesting.

## Author Contributions

S.A.S. designed, executed the study, analyzed the data, and wrote the manuscript. M.T.K. designed the outlines of the course, recruited the participants, and appointed the course coordinators. Both S.A.S. and M.T.K. read and approved the final manuscript.

## Ethics Statement

Ethical approval was sought and obtained from the Head of Veterinary Medicine, University of Buea, Southwest Cameroon.

## Consent

The authors have nothing to report.

## Conflicts of Interest

The authors declare no conflicts of interest.

## Data Availability

The data that supports the findings of this study are available in the Supporting Information of this article.
